# Ocrelizumab zur Behandlung der Multiplen Sklerose

**DOI:** 10.1007/s00115-020-00937-6

**Published:** 2020-06-10

**Authors:** Jonas Graf, Philipp Albrecht, Norbert Goebels, Orhan Aktas, Hans-Peter Hartung

**Affiliations:** Klinik für Neurologie, Universitätsklinikum Düsseldorf, Medizinische Fakultät, Heinrich-Heine-Universität, Moorenstraße 5, 40225 Düsseldorf, Deutschland

**Keywords:** B‑Zell-depletierende monoklonale Antikörper, B‑Zellen, Immunsuppression, Klinische Neuroimmunologie, Neuroinflammation, B cell depleting monoclonal antibodies, B cells, Immunosuppression, Clinical neuroimmunology, Neuroinflammation

## Abstract

Ocrelizumab ist ein monoklonaler Antikörper, der sich gegen das Differenzierungsantigen CD20 richtet und zu einer effektiven längerfristigen Depletion von Lymphozyten, insbesondere von B‑Zellen, führt. Unlängst publizierte Phase-3-Studien belegen, dass Ocrelizumab sowohl bei der Behandlung der schubförmigen als auch der primär progressiven Multiplen Sklerose (MS) wirksam ist. Darauf basierend wurde Ocrelizumab als erstes Medikament zur Behandlung der primär chronisch-progredienten MS zugelassen. Um diesen Durchbruch besser in den Kontext des heutigen MS-Therapiekanons einordnen zu können, lohnt sowohl ein Blick zurück auf die Entwicklung der antikörpervermittelten CD20-Depletion als auch auf die der Zulassung zugrunde liegenden Studien sowie deren Extensionsphasen. Diese Übersichtsarbeit diskutiert die verfügbaren Daten zur Wirksamkeit und Sicherheit der langfristigen B‑Zell-Depletion bei MS-Patienten und erörtert den aktuellen Kenntnisstand zur Rolle von B‑Lymphozyten in der Immunpathogenese der MS.

## Abstract (en)

Ocrelizumab is a monoclonal antibody directed against the differentiation antigen CD20, which leads to an effective long-term depletion of lymphocytes, in particular B cells. Recently published phase 3 studies confirmed that ocrelizumab is effective in the treatment of both relapsing multiple sclerosis (RMS) and primary progressive multiple sclerosis (PPMS). Based on these results, ocrelizumab was the first drug to be approved for primary chronic progressive MS. To place this therapeutic breakthrough in the context of the current MS therapeutic landscape, it is worthwhile taking a look back at the development of antibody-mediated CD20 depletion, the studies underlying the approval of ocrelizumab and their open extension phases. This review article discusses the available data on the efficacy and safety of long-term B‑cell depletion in MS patients and reviews current knowledge on the role of B‑lymphocytes in the immunopathogenesis of MS.

## Hintergrund

**Figure 1:**
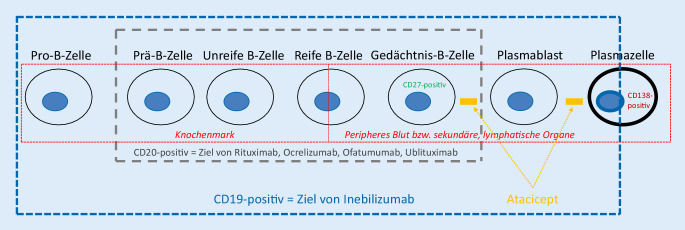

Die Multiple Sklerose (MS) ist mit weltweit ca. 2,5 Mio. Betroffenen die häufigste immunvermittelte, chronisch-entzündliche Erkrankung des zentralen Nervensystems (ZNS). Der Krankheitsverlauf ist entweder schubförmig (RMS) – mehrheitlich im Verlauf mit Übergang in die sekundär progrediente Form (SPMS) oder primär progredient [1]. Trotz jahrzehntelanger Forschung sind die eigentlichen Ursachen der Erkrankung nicht geklärt. Derzeitigen Hypothesen zufolge handelt es sich um eine komplexe Genese, zu der sowohl genetische als auch immunologische und umweltbedingte Faktoren beitragen. Eine Heilung der MS ist bis dato nicht möglich. In den letzten zwei bis drei Dekaden gab es jedoch eine erfreuliche Entwicklung zahlreicher immunmodulatorischer Therapieoptionen [1–3]. Mechanistisch neuartig ist die Depletion von CD20-positiven Zellen, ein Therapieprinzip, das sich bei anderen Autoimmunerkrankungen in Form von Rituximab bereits länger bewährt hat [4, 5]. Das CD20-Molekül ist ein Mitglied der „membrane spanning 4A family“ und wird vom *MS4A1*-Gen auf Chromosom 11 kodiert [6]. Mittels CD20-Depletion konnte nicht nur der schubförmige (RMS bzw. RRMS), sondern erstmals auch der primär chronisch-progrediente Krankheitsverlauf (PPMS) positiv beeinflusst werden. Abb. 1 veranschaulicht die Entwicklungsstadien der B‑Zell-Linie, die von einer gegen das CD20-Differenzierungsantigen gerichteten Therapie betroffen sind (adaptiert aus [7]). Dieser therapeutische Ansatz ist hoch effektiv und es spricht für sich, dass sich aktuell Nachfolgepräparate mit ähnlichem Wirkprofil [8] in der klinischen Entwicklung befinden bzw. gerade zugelassen wurden (z. B. der oral applizierte Bruton-Tyrosin-Kinase-Hemmer Evobrutinib [9] NCT04032158 und die monoklonalen Antikörper Ofatumumab [10] NCT02792218 bzw. NCT02792231, Ublituximab NCT03277261 bzw. NCT03277248 und NCT04032171). Die Ergebnisse der Ofatumumab-Zulassungsstudien wurden 2019 auf dem ECTRIMS-Kongress präsentiert. Ofatumumab führte zu einer relativen Risikoreduktion von 50,5 % (ASCLEPIOS I) bzw. 58,5 % (ASCLEPIOS II) der auf ein Jahr umgerechneten Schubrate im Vergleich zu Teriflunomid [11]. Die Substanz wurde gut vertragen.

Interessanterweise führen niedrig dosierte intrathekale Gaben von Rituximab zu einer kompletten CD20-Depletion im peripheren Blut [12], jedoch zu keiner vollständigen CD20-Depletion im ZNS [13]. Diese therapeutischen Entwicklungen haben dazu beigetragen, unsere Konzepte der pathophysiologischen Rolle der Immunzellen in der MS wesentlich zu wandeln [14].

**Figure 2:**
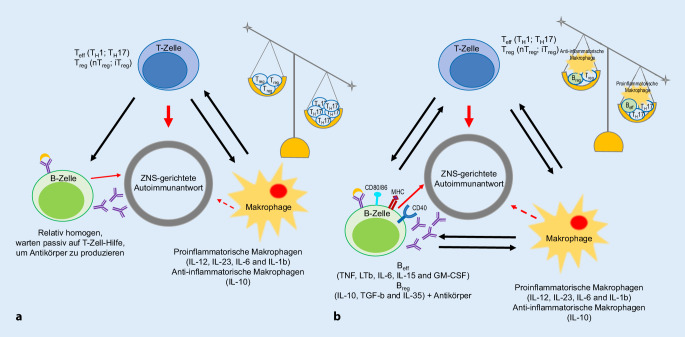

Aktuell geht man davon aus, dass B‑Zellen – und nicht mehr ausschließlich oder dominant T‑Zellen – eine zentrale Bedeutung in der MS zukommt (Abb. 2, adaptiert aus [14]). Tab. 1 fasst die Rolle von B‑Zellen in der Pathophysiologie der MS zusammen.

**Table 1:** 

Intrathekale Produktion von Immunglobulinen (oligoklonale Banden)
Produktion von Antikörpern gegen Myelinbestandteile in Blut und Liquor
B‑Zell- und Komplementablagerungen in Hirnläsionen
Meningeale B‑Zell-Aggregate bei SPMS
Expandierte Plasmablasten in Blut und Liquor
Antigenpräsentation, Zytokinproduktion, Förderung des „homing“ autoreaktiver T‑Zellen ins ZNS
Induktion und Regulation der Proliferation autoreaktiver, proinflammatorischer T‑Zellen (u. a. T_H_17-Zellen)
Induktion der Apoptose von Neuronen und Oligodendrozyten

*SPMS* Sekundär progrediente Multiple Sklerose

## Rituximab bei RRMS und PPMS: Fallberichte und -serien

Die ersten Berichte über den Einsatz von CD20-Depletion bei Patienten mit Multipler Sklerose stammen aus den Jahren 2005 und 2006. In einer ersten Fallserie von PPMS-Patienten wurde zunächst das B‑Zell-Muster sowohl im peripheren Blut als auch im Liquor vor und nach Gabe von Rituximab untersucht. Erwartungsgemäß depletierte Rituximab B‑Zellen im peripheren Blut rasch, effektiv und längerfristig, die Wirkung auf B‑Zellen im Liquor war jedoch gering ausgeprägt. Auch konnte gezeigt werden, dass die Kinetik der B‑Zell-Repopulation individuell bei Patienten deutlich schwanken kann [15]. Diese Ergebnisse stehen teilweise im Widerspruch zu einer weiteren Fallserie, bei der Rituximab als Zusatztherapie bei RRMS-Patienten verabreicht wurde: Bei diesen Patienten zeigte sich ein Abfall von sowohl B‑ als auch T‑Lymphozyten im Liquor [16]. Bei einem anderen RRMS-Patienten mit fulminantem Krankheitsverlauf kam es nach intravenöser Gabe von Rituximab nicht nur zum Stillstand der Erkrankung, sondern auch zu Besserungen des klinischen Funktionsstatus [17–19].

## Rituximab bei RRMS: Phase-1- und -2-Studien

Eine nichtkontrollierte „Open-label“-Phase-1-Studie mit 26 RRMS-Patienten belegte ein günstiges Sicherheitsprofil von Rituximab über 72 Wochen. Der Antikörper verminderte die Anzahl neu aufgetretener Hirnläsionen zwischen Woche 4 und 72 und reduzierte die Schubrate im Vergleich zum Jahr vor Studienbeginn [20]. Die anschließende, doppelblinde, placebokontrollierte Phase-2-Studie (HERMES, Tab. 2) konnte dokumentieren, dass Rituximab nicht nur im primären magnetresonanztomographischen (MRT-)Endpunkt, sondern auch in den sekundären klinischen Zielkriterien gegenüber Placebo überlegen war [21].

**Table 2:** 

Studie	Primärer Endpunkt *Ergebnis*	Sekundärer Endpunkt *Ergebnis*
Phase-2-RRMS Hauser et al. [21] (HERMES) *n* = 104 Patienten	Summe der Anzahl der gadoliniumpositiven T1-Läsionen in Serien-MRTs des Gehirns in den Wochen 12, 16, 20, 24 *→ Rituximab Placebo überlegen*	Anteil der Patienten mit Schüben Auf das Jahr umgerechnete Schubrate Absolute Anzahl von neuen gadoliniumpositiven T1-Läsionen in Serien-MRTs des Gehirns in den Wochen 12, 16, 20, 24 und Änderung des T2-Läsionsvolumens im Vergleich zum Ausgangswert *→ Rituximab Placebo überlegen*
Phase-2/3-PPMS Hawker et al. [22] (OLYMPUS) *n* = 439 Patienten	Zeit bis zum Einsetzen von CDP Prozentsatz der Patienten mit CDP *→ Kein Hinweis für einen signifikanten Unterschied*	Änderung des absoluten T2-Läsionsvolumens in Woche 96 im Vergleich zum Ausgangswert Änderung des Hirnvolumens in Woche 96 im Vergleich zum Ausgangswert *→ Patienten, die Rituximab erhalten haben, hatten eine signifikant geringere Zunahme an T2-Läsionsvolumen*

*MRT* Magnetresonanztomographie, *CDP* „confirmed disability progression“ (bestätigte Krankheitsprogression)

## Rituximab bei PPMS: Phase-2/3-Studie

Aufgrund der überraschend guten Wirksamkeit der CD20-Depletion bei Patienten mit schubförmiger Multipler Sklerose wuchs die Hoffnung, dass dieser therapeutische Ansatz ebenfalls einen positiven Einfluss auf den primär chronisch-progredienten Krankheitsverlauf haben könnte. Die randomisierte, doppelblinde, placebokontrollierte Phase-2/3-OLYMPUS-Studie bei PPMS (Tab. 2) verfehlte jedoch den primären klinischen Endpunkt: Es konnte kein signifikanter Unterschied in der Wirksamkeit auf die bestätigte Behinderungsprogression nachgewiesen werden. Doch der sekundäre radiologische Endpunkt, die signifikante Reduktion der T2-Läsionslast, wurde erreicht und Subgruppenanalysen zeigten, dass insbesondere jüngere Patienten, die im MRT aktive entzündliche Läsionen aufweisen, auch klinisch von einer Therapie mit Rituximab profitierten [22, 23].

## Ocrelizumab: RRMS-Phase-2-, RRMS-Phase-3- und PPMS-Phase-3-Studien sowie Folgestudien nach Zulassung

In den folgenden Studien zur Wirksamkeit der CD20-Depletion bei MS-Patienten wurde nun nicht mehr der chimäre monoklonale Antikörper Rituximab, sondern der strukturell humanisierte Antikörper Ocrelizumab verwendet (Tab. 3). Die randomisierte, doppelt verblindete Phase-2-Studie mit Interferon‑β_1a_ (IFN‑β_1a_ 30 μg pro Woche intramuskulär) als aktivem Komparator zeigte die Überlegenheit von Ocrelizumab hinsichtlich des radiologisch definierten primären Endpunktes (Anzahl der gadoliniumaufnehmenden T1-Läsionen im der zerebralen MRT). Hinsichtlich der Nebenwirkungen zeigte sich kein relevanter Unterschied zwischen den einzelnen Gruppen [24].

**Table 3:** 

Studie	Primärer Endpunkt *Ergebnis*	Sekundärer Endpunkt *Ergebnis*
Phase-2-RRMS Kappos et al. [24]	Anzahl der gadoliniumpositiven T1-Läsionen zwischen Woche 12 und 24 *→ Ocrelizumab Placebo überlegen*	Auf das Jahr umgerechnete Schubrate Prozentsatz der schubfreien Patienten Änderung des absoluten T2-Läsionsvolumens Anzahl der neuen gadoliniumpositiven T1-Läsionen zwischen Woche 4 und 24 Anzahl der gadoliniumpositiven T1-Läsionen zwischen Woche 4 und 24 *→ Ocrelizumab Placebo in allen Punkten überlegen außer Prozentsatz der schubfreien Patienten und Änderung des absoluten T2-Läsionsvolumens*
Phase-3-RMS Hauser et al. [25] (OPERA I und II)	Auf das Jahr umgerechnete Schubrate *→ Ocrelizumab IFN‑β*_*1a*_* überlegen*	Zeit bis zum Einsetzen von CDP Anzahl der gadoliniumpositiven T1-Läsionen Anzahl der neuen und/oder vergrößerten T2-Läsionen Prozentsatz der Patienten mit CDI Anzahl der T1-Läsionen Änderung des MSFC im Vergleich zum Ausgangswert Prozentsatzänderung des Hirnvolumens Änderung des Short Form Health Survey-36 (SF-36) Physical Component Summary (PCS) im Vergleich zum Ausgangswert Prozentsatz der Patienten mit NEDA *→ Ocrelizumab überlegen in allen sekundären Endpunkten außer MSFC und SF-36 in OPERA I und überlegen in allen sekundären Endpunkten außer CDI und *Prozentsatzänderung des Hirnvolumens* in OPERA II*
Phase-3-PPMS Montalban et al. [26] (ORATORIO)	Zeit bis zum Einsetzen von anhaltender CDP für mindestens 12 Wochen *→ Ocrelizumab Placebo überlegen*	Zeit bis zum Einsetzen von anhaltender CDP für mindestens 24 Wochen Prozentsatzänderung des T25-FW im Vergleich zum Ausgangswert Prozentsatzänderung des absoluten T2-Läsionsvolumens im Vergleich zum Ausgangswert Prozentsatzänderung des Hirnvolumens Änderung im Physical Component Summary Score (PCS) und SF-36 Health Survey Prozentsatz der Patienten mit mindestens einem unerwünschten Ereignis *→ Ocrelizumab überlegen in Bezug auf Zeit bis zum Einsetzen von anhaltender CDP für mindestens 24 Wochen, Prozentsatzänderung des T25-FW im Vergleich zum Ausgangswert*; *Prozentsatzänderung des absoluten T2-Läsionsvolumens im Vergleich zum Ausgangswert, Prozentsatzänderung des Hirnvolumens*

*T25FW* Timed 25-Foot Walk, *MSFC* Multiple Sclerosis Functional Composite, *NEDA* „no evidence of disease activity“ (kein Anhalt für Krankheitsaktivität), *CDP* „confirmed disability progression“ (bestätigte Krankheitsprogression), *CDI* „confirmed disability improvement“ (bestätigte Verbesserung des Behinderungsgrads), *IFN* Interferon

Zur Zulassung von Ocrelizumab bei RMS und PPMS führten die anschließenden Phase-3-Studien, die alle ihre primären klinisch definierten Endpunkte erreichten: die beiden identisch designten Studien OPERA I und II zu Ocrelizumab vs. Interferon‑β_1a_ (intramuskulär) bei RMS [25] sowie die Studie ORATORIO zu Ocrelizumab vs. Placebo bei früher PPMS [26] definiert über Alter (18 bis 55 Jahre) und Erkrankungsdauer (<15 Jahre bei EDSS >5,0 bzw. <10 Jahre bei EDSS <5,0).

In den OPERA-Zwillingsstudien bei RMS reduzierte Ocrelizumab die jährliche Schubrate gegenüber IFN‑β_1a_ um 46 % bzw. 47 % (jeweils *p* < 0,0001). Zudem wurden alle sekundären Endpunkte erreicht, darunter die Reduktion der Behinderungsprogression bzw. die Besserung der Behinderung (jeweils mit Bestätigung nach 12 und 24 Wochen) und magnetresonanztomographische Wirksamkeitskriterien, wobei die Reduktion der prozentualen Veränderung des Hirnvolumens nur in OPERA I statistisch signifikant war. Den Status NEDA über 2 Jahre erreichten in beiden Studien 48 % der Patienten in der Ocrelizumab-Gruppe gegenüber 29 % bzw. 25 % unter der aktiven Vergleichstherapie.

Eine jüngst veröffentlichte Post-hoc-Analyse belegte eine bestätigte Verbesserung der Armfunktion, erfasst in 12-wöchigen Abständen mit dem 9 Hole Peg Test (9HPT). In der Intention-to-treat-Analyse war auch der Anteil von Patienten mit bestätigter Verschlechterung im 9HPT geringer in der Ocrelizumab-behandelten Gruppe [27].

In einer kürzlich veröffentlichen Analyse der Krankheitsprogression in den OPERA-Studien, zeigt sich, dass in der gesamten RMS-Population der größte Anteil der erworben Behinderung schubunabhängig erfolgt [28].

In der 120-wöchigen PPMS-Studie ORATORIO erreichte Ocrelizumab sowohl den primären Endpunkt (Reduktion des Risikos einer nach 12 Wochen bestätigten Behinderungsprogression) als auch die sekundären Endpunkte. Der Anteil der Patienten mit bestätigter Krankheitsprogression im EDSS-Score nach 12 Wochen war gegenüber Placebo um 24 % reduziert. Subanalysen der Handfunktion (9HPT) und Gehfähigkeit (T25FW) bestätigten die Überlegenheit von Ocrelizumab in diesen Teilbereichen der motorischen Funktion [26].

Es ist zu erwähnen, dass in der PPMS-Studie nur Patienten eingeschlossen wurden, die eine relativ kurze Erkrankungsdauer – definiert über Alter (18 bis 55 Jahre) und Erkrankungsdauer (Symptomdauer <15 Jahre bei Patienten mit einem EDSS-Wert von >5,0 oder <10 Jahre bei Patienten mit einem EDSS von <5,0 zum Zeitpunkt des Screenings) – hatten.

Das Volumen von T2-Hirnläsionen nahm in der Ocrelizumab-Gruppe um 3,4 % ab, während es unter Placebo um 7,4 % anstieg. Die Anzahl neuer T2-Läsionen war unter Ocrelizumab gegenüber Placebo um 92 % reduziert [26]. Auch die Abnahme des Gehirnvolumens war in der Gruppe mit aktiver Therapie signifikant vermindert. Subgruppenanalysen zufolge war das Ansprechen auf Ocrelizumab nicht von der Präsenz gadoliniumaufnehmender Läsionen zu Beginn der Studie abhängig [26].

Bei Neuromyelitis-optica-Spektrumerkrankungen (NMOSD), einer Gruppe schubförmig verlaufender chronisch-entzündlicher ZNS-Erkrankungen mit pathognomonischer Astrozytopathie, konnte gezeigt werden, dass eine B‑Zell-Repopulation mit einem Anstieg der Schubrate assoziiert ist [29]. Inwiefern sich dieser Zusammenhang auf die RMS übertragen lässt, ist bislang allerdings unklar.

In den Zulassungsstudien kam es bei 20,7 % der RMS-Patienten und bei 26,3 % der PPMS-Patienten zu einem Abfall der absoluten Lymphozyten unterhalb des unteren Normalwertes [30]. Die Mehrheit der Patienten entwickelte eine Grad-1- oder -2-Lymphopenie, die Rate der Grad-3-Lymphopenien lag bei 1 % und bereits nach 2 Wochen ließen sich keine CD19-positiven Zellen mehr im Blut nachweisen [22, 26, 30]. Nach 2,5 Jahren (Median 72 Wochen) Ocrelizumab-Therapiepause hat sich bei 90 % der Patienten die Lymphozytenpopulation erholt [30]. Im Vergleich dazu hat sich die Lymphozytenpopulation in der Rituximab-Phase-2/3-Studie (OLYMPUS) nach 48 Wochen bei 35 % der Patienten erholt [22]. In den Folgestudien nach Marktzulassung am 12.01.2018 ([30]; Indikationen siehe Tab. 4; Anwendungsschema siehe Tab. 5) konnte der Nutzen von Ocrelizumab weiter bestätigt werden: 66,4 % der RMS-Patienten unter Ocrelizumab und 24,3 % der Patienten unter Interferon‑β_1a_ zeigten keinen Hinweis für klinische oder radiologische Krankheitsaktivität („no evidence of disease activity“, NEDA; [31]). Da direkte Vergleichsstudien von Ocrelizumab gegen andere MS-Therapien fehlen, wurde eine Metaanalyse durchgeführt, die zeigte, dass der Nutzen einer Ocrelizumab-Therapie insbesondere bei Patienten mit hochaktiver RMS gegeben ist [32]. Ferner lieferten zahlreiche retrospektive Analysen und eine Subgruppenanalyse Hinweise dafür, dass Rituximab sowohl effektiv bei aggressiver RMS bzw. progressiver MS sein kann [33–39] als auch den MS-Therapien der 1. Generation (i.e. Interferon‑β und Glatirameracetat) überlegen ist [40, 41]. Ob Rituximab allerdings eine gleichwertige Alternative zu Ocrelizumab darstellt, bleibt in Abwesenheit einer Kopf-an-Kopf-Phase-III-Studie Gegenstand von Diskussionen [42–45]. Direkte Vergleichsstudien zwischen Rituximab und anderen Therapieoptionen wären sinnvoll, um diese Wissenslücken zu schließen [46].

**Table 4:** 

Ocrelizumab ist angezeigt zur Behandlung erwachsener Patienten mit schubförmiger Multipler Sklerose (RMS) mit aktiver Erkrankung, definiert durch klinischen Befund oder Bildgebung
Ocrelizumab ist angezeigt zur Behandlung erwachsener Patienten mit früher primär progredienter Multipler Sklerose (PPMS), charakterisiert anhand der Krankheitsdauer und dem Grad der Behinderung sowie mit Bildgebungsmerkmalen, die typisch für eine Entzündungsaktivität sind

**Table 5:** 

		Infusionsmenge Ocrelizumab	Infusionsgeschwindigkeit und -dauer
Initialdosis (600 mg) aufgeteilt auf 2 Infusionen	Infusion 1	300 mg in 250 ml	Infusionseinleitung mit einer Geschwindigkeit von 30 ml/h über 30 min Die Geschwindigkeit kann in Schritten von 30 ml/h alle 30 min bis auf einen Höchstwert von 180 ml/h gesteigert werden Die Infusionsdauer sollte jeweils ca. 2,5 h betragen
	Infusion 2 (2 Wochen später)	300 mg in 250 ml	
Folgedosen (600 mg) einmal alle 6 Monate	Einmalinfusion	600 mg in 500 ml	Infusionseinleitung mit einer Geschwindigkeit von 40 ml/h über 30 min Die Geschwindigkeit kann in Schritten von 40 ml/h alle 30 min bis auf einen Höchstwert von 200 ml/h gesteigert werden Die Infusionsdauer sollte jeweils ca. 3,5 h betragen

Um das Ansprechen der PPMS-Patienten auf eine Immuntherapie besser charakterisieren zu können, wird seit kurzem der Summenparameter mit dem Akronym NEPAD – „no evidence of progression or active disease“ – verwendet. In der ORATORIO-Studie erhöhte Ocrelizumab den Anteil der PPMS-Patienten mit NEPAD nach 120 Wochen im Vergleich zu Placebo um das Dreifache [47]. Eine Analyse der gepoolten Daten aus den Phase-2- und -3-Studien legt nahe, dass Ocrelizumab die MRT-Läsionsaktivität innerhalb von 4 Wochen und klinische Krankheitsaktivität innerhalb von 8 Wochen effektiv unterdrückt [48]. Eine MR-Spektroskopie-Studie mit sequenzieller Messung über 96 Wochen eines Markers des „neuronal-myelin coupling“ (NAA, tCr, tCho und NAA/tCr) zeigt, dass Ocrelizumab das Ausmaß einer zerebralen Gliose im Vergleich zu Interferon‑β reduziert [49]. Die klinische Progression scheint bei PPMS-Patienten mit chronischer Läsionsaktivität in der weißen Substanz assoziiert zu sein [50].

## Ocrelizumab: Nebenwirkungen

### Akute Infusionsreaktionen

Als relevante Nebenwirkungen traten unter Ocrelizumab gegenüber Placebo häufiger akute Infusionsreaktionen und Infektionen auf. Die Häufigkeiten der schwerwiegenden Nebenwirkungen in den Zulassungsstudien sind in Tab. 6 zusammengefasst. Weitere laufend aktualisierte Daten – insbesondere zu Infektionen und Neoplasien – werden regelmäßig auf Kongressen vorgestellt und ergaben bislang keine neuen Sicherheitsaspekte [51].

**Table 6:** 

Studie	Anzahl	Davon Infektionen
OPERA I	28 (6,9 %)	5 (1,2 %)
OPERA II	29 (7,0 %)	6 (1,4 %)
ORATORIO	99 (20,4 %)	30 (6,2 %)

Eine retrospektive Studie konnte zeigen, dass das Auftreten von infusionsassoziierten Reaktionen durch die zusätzliche Gabe von Histaminantagonisten und oraler Flüssigkeitszufuhr deutlich reduziert werden kann [52].

### Malignome

Im klinischen Studienprogramm wurde bei Patienten, die mit Ocrelizumab behandelt wurden, eine verglichen mit den Kontrollgruppen höhere Anzahl maligner Erkrankungen (einschließlich Mammakarzinom) beobachtet [53, 54].

In der 2:1 randomisierten Zulassungsstudie zur PPMS wurden unter Ocrelizumab (486 Patienten) in der placebokontrollierten Phase 11 Neoplasien verzeichnet, (Mammakarzinom 4, Basalzellkarzinom 3, endometrioides Adenokarzinom 1, anaplastisches großzelliges Lymphom 1, fibröses Histiozytom 1, Pankreaskarzinom 1), während in der Placebogruppe (239 Patienten) in der kontrollierte Studienphase 2 Neoplasien auftraten (Basalzellkarzinom 1, zervikales Adenokarzinom in situ 1; [26]).

Bei den RMS-Patienten unter Ocrelizumab traten in den 2‑jährigen kontrollierten Phasen der OPERA-Studien insgesamt 4 maligne Erkrankungen auf (Mammakarzinom 2; Nierenzellkarzinom 1, malignes Melanom 1) und in der mit IFN‑β_1a_ behandelten Gruppe 2 (Mantelzell-Lymphom 1 und pulmonales Plattenepithelkarzinom 1; [25]).

Jährlich aktualisierte Analysen (aktueller Stand Januar 2019) zur Inzidenzrate von malignen Erkrankungen (ohne nichtmelanotische Hautmalignome) bei den weiter beobachteten Patienten des klinischen Studienprogramms zu Ocrelizumab zeigen, dass die alters- und geschlechtsstandardisierten Raten pro 100 Patientenjahre in der exponierten Population über die Zeit stabil blieben und keinen zeitabhängigen Expositionseffekt zeigen. Des Weiteren ergaben Vergleiche der standardisierten Inzidenzraten für alle maligne Erkrankungen und für Mammakarzinome mit der US-amerikanischen SEER-Datenbank (Allgemeinbevölkerung) und dem dänischen MS-Register, dass die beobachteten Inzidenzraten im gesamten bisherigen Beobachtungszeitraum innerhalb der epidemiologischen Referenzbereiche lagen [55].

### Infektionen

Fallberichte weisen auf das Risiko einer Hepatitisreaktivierung unter CD20-Depletion hin [56, 57], daher ist eine Risikostratifizierung vor Therapiebeginn sinnvoll [58]. Ferner wurden als Risiken eine Late-onset-Neutropenie [59, 60] und das Auftreten tumefaktiver, demyelinisierender Läsionen beschrieben [61]. Es sind wenige Fälle einer „carry-over“ progressiven multifokalen Leukenzephalopathie (PML) bekannt geworden. Diese traten nach vorangegangener Therapie mit anderen Immunmodulatoren mit bekannt erhöhtem PML-Risiko auf (7 Fälle Stand Ende 2019) [62, 63]. Aktuell ist eine nichtinterventionelle Studie geplant, die das Langzeitrisikoprofil von Ocrelizumab besser erfassen soll [64]. Die Ergebnisse dieser Studie in Deutschland fließen auch in globale Sicherheitsregister ein [65].

Dieser Kontext ist ähnlich wie bei den Fällen einer PML in Assoziation mit dem Vorläuferpräparat Rituximab in anderen Nicht-MS-Indikationen, wie z. B. rheumatoider Arthritis [66]. Die Auswertung eines Rituximab-Therapieregisters ergab keine neuen Aspekte hinsichtlich des Nebenwirkungsprofils von CD20-Depletion [67]. Es gibt Hinweise dafür, dass ein sekundärer Immunglobulinmangel das Auftreten schwerer Infektionen begünstigen kann [68]. In einem Review über das Risiko einer Hypogammaglobulinämie bzw. Infektion unter Rituximab konnten jedoch keine klaren Risikofaktoren herausgearbeitet werden [69]. Wie unter Rituximab sollte unter Ocrelizumab regelmäßig der IgG-Spiegel kontrolliert werden [70]. Dies ist insbesondere relevant, da die Infektionsrate unter CD20-Depletion bei MS-Patienten am höchsten zu sein scheint [71].

Bei einer 78 Jahre alten Patientin, mit niedrigen CD4^+^- und CD8^+^-Lymphozytenzahlen vor Therapiebeginn und vermuteter Immunseneszenz wurde eine PML unter Ocrelizumab ohne Vorexposition gegenüber anderen immunologischen Therapien gemeldet [72].

Bezüglich anderer infektiöser Komplikationen wurden einzelne Fallberichte von Patienten publiziert, die unter einer Ocrelizumab-Behandlung eine Herpes-simplex-Typ-2-Enzephalitis [73], eine Parvovirus-B19-Infektion [74] und in 2 Fällen eine Meningitis entwickelten. In einem Fall verlief die Meningitis aseptisch, bei der anderen konnte eine vermutete Borreliengenese nicht verifiziert werden [75]. Ein Fallbericht legt nahe, dass Ocrelizumab zwar B‑Zellen depletiert, jedoch nicht die T‑Zell-Immunantwort auf Varizella Zoster beeinflusst [76].

Kürzlich wurde unter Ocrelizumab das Auftreten einer Endokarditis berichtet [77].

Die Therapie mit Ocrelizumab ist wahrscheinlich nicht mit einem erhöhten Risiko einer Tuberkuloseinfektion assoziiert [78].

Es ist zu beachten, dass es bei RMS-Patienten unter Ocrelizumab im Vergleich zu Interferon‑β_1a_ mehr Infektionen mit Varizella-Zoster-Viren (*n* = 17 vs. 8 [25]) auftraten und bei PPMS-Patienten unter Ocrelizumab im Vergleich zu Placebo mehr Episoden mit oralem Herpes simplex (2,3 % vs. 0,4 %; [25]). In der Zusammenschau sind regelmäßige Verlaufskontrollen bei MS-Patienten unter CD20-depletierenden Therapien sinnvoll [79].

Obwohl sowohl eine retrospektive Analyse von Patienten mit neuroimmunologischen Erkrankungen unter 7‑jähriger Rituximab-Therapie [80] als auch eine Metaanalyse von Patienten mit rheumatoider Arthritis [81] ein günstiges Sicherheitsprofil bestätigen konnten, ist der Einfluss einer Langzeit-B-Zell-Depletion auf Malignom- und Infektionsrisiken nicht vollständig charakterisiert [82, 83]. Für Ocrelizumab liegen aktuell aus klinischen Studien Daten von Patienten mit bis zu 6 Jahren Exposition vor, mit einer Gesamtexposition von über 14.000 Patientenjahren (Stand Mai 2019; [55]).

### Schwangerschaften unter CD20-gerichteter Therapie

Es zeigten sich keine klaren Sicherheitssignale (i.e. Spontanaborte oder kindliche Fehlbildungen) bei Schwangeren, die in den ersten 6 Schwangerschaftsmonaten eine CD20-Depletion mittels Rituximab erfahren haben [84]. Die Auswertung der bisher dokumentierten Schwangerschaften mit Ocrelizumab-Exposition ergab keine Auffälligkeiten [85]. Weitere Daten werden im Ocrelizumab-Schwangerschaftsregister erhoben [86].

Die Datenlage bezüglich intrathekaler Applikation von CD20-depletierenden monoklonalen Antikörpern ist gering [87]. Ferner ist hervorzuheben, dass es bei RMS-Patienten unter Ocrelizumab im Vergleich zu Interferon‑β_1a_ zu mehr Varizella-Zoster-Infektionen (17 vs. 8 [25]) und bei PPMS-Patienten unter Ocrelizumab im Vergleich zu Placebo zu mehr oralen Herpesinfektionen (2,3 % vs. 0,4 %; [26]) gekommen ist.

## Pathomechanistische Aspekte der Rolle von B-Zellen in der MS

Die rasch einsetzenden tiefgreifenden Effekte einer CD20-B-Zell-gerichteten Therapie haben zu einer Neubewertung der humoralen Immunantwort bei MS geführt. Nach ursprünglicher Vorstellung würde eine B‑Zell-Depletion vermutlich wesentlich über eine Verminderung der Produktion von Autoantikörpern wirken.

Die Wirkungsweise von CD20-Depletion bei MS und den autoimmunen Tiermodellen ist allerdings bisher nicht vollständig verstanden [88]. Die aktuelle Datenlage hinsichtlich der B‑Zell-Rolle bei MS wurde rezent mehrfach zusammengefasst [7, 14, 89–92] und die Interaktion von B‑ und T‑Zellen diskutiert [93]. Die Effektivität der B‑Zell-Depletion bei MS unterstützt die Hypothese, dass mit Epstein-Barr-Viren latent infizierte B‑Zellen eine wichtige Rolle bei der Pathogenese der MS spielen könnten [94, 95]. Aus tierexperimentellen Studien gibt es Hinweise, dass eine CD20-Depletion die Aktivierung von Mikrogliazellen und die Rekrutierung von T‑Lymphozyten reduziert [96]. Die pathogene CD40-vermittelte NF-κB-Aktivierung in B‑Zellen ist bei MS-Patienten verstärkt [97]. Zusätzlich werden B‑Zell Aggregate in lymphfollikelähnlichen Strukturen im Subarachnoidalraum, insbesondere bei Patienten mit sekundär chronisch-progredienter Verlaufsform, als erkrankungsrelevant diskutiert [98, 99]. Eine tierexperimentelle Studie konnte diesbezüglich zeigen, dass eine intrathekale Gabe von Anti-CD20-Antikörpern zu einer relevanten Depletion von B‑Zellen in etablierten ZNS-Läsionen führt [100]. Des Weiteren gibt es Hinweise dafür, dass es bei MS-Patienten einen Transit zwischen dem ZNS und der Peripherie von insbesondere immunaktiven B‑Zellen gibt, die einen Immunglobulinklassenwechsel erfahren haben [101], und dass B‑Zellen von MS-Patienten vermehrt Antigene präsentieren [102]. Diese Immunzellen müssen dabei mittels komplexer, mehrschrittiger Kaskaden Immunbarrieren überwinden [103], um dort klonal zu expandieren [104] und spezifische Immunglobuline zu produzieren [105] und Lymphangiogenese zu fördern [106]. Die Reifung der ZNS-ständigen B‑Zellen bei MS-Patienten erfolgt nach aktuellem Wissenstand in den drainierenden zervikalen Lymphknoten [107]. B‑Zellen von RRMS-Patienten sind in der Lage, komplement- und immunglobulinunabhängige Faktoren zu produzieren, die in vitro toxisch für Neurone und Oligodendrozyten sind [108]. Bei Patienten mit Myelitis als klinisch isoliertem Syndrom, die sich somit im Anfangsstadium einer möglichen MS befinden, konnte sowohl eine expandierte und mutierte Plasmablastensubgruppe [109] als auch eine bestimmte Verteilung von Interleukin-6/-10-produzierenden B‑Lymphozyten differenziert werden [110], was im Einklang mit dem aktuellen Verständnis über die Rolle der Zytokine bei autoimmunen Erkrankungen steht [111]. Somit scheint es, dass B‑Zellen sowohl zu Beginn als auch bei der Aufrechterhaltung der MS-Erkrankung eine wichtige Rolle spielen [112]. Wichtig dabei scheint die Regulierung des „Macrophage-migration-inhibitory-factor“(MIF)-Signalwegs zu sein, da ein supprimiertes CD74 und ein hochreguliertes CXCR4 mit einer frühen MS-Diagnose assoziiert sind [113]. Ferner wird diskutiert, dass nicht alle B‑Lymphozyten pathophysiologisch relevant sind, sondern insbesondere eine bestimmte Subgruppe, und zwar „Granulocyte-macrophage-colony-stimulating-factor“(GM-CSF)-exprimierende humane B‑Gedächtnis-Zellen [114], bzw. verschiedene B‑Lymphozyten-Subgruppen im Liquor entscheidend sind für den Erkrankungsphänotyp [115]. Basierend auf den Erkenntnissen über die phasenabhängige Oberflächenexpression von Differenzierungsantigenen in der B‑Zell-Reifung und der Diskussion einer aktuellen Phase-2/3-Studie von Inebilizumab, ein gegen CD19 gerichteter monoklonaler Antikörper, bei NMOSD [116] muss zwischen CD20- und CD19-Depletion unterschieden werden (Abb. 1): CD20 wird nicht auf der Oberfläche von Plasmazellen exprimiert, wohingegen CD19 auf der Mehrheit von Plasmazellen in sekundären lymphatischen Organen (z. B. Milz, Tonsillen), auf allen Plasmazellen im Blut und bei mehr als 50 % der Plasmazellen im Knochenmark nachgewiesen werden kann [117, 118]. Zusätzlich muss beachtet werden, dass CD19 nur von Plasmazellen, CD20 jedoch auch von einer Untergruppe CD-positiver T‑Zellen exprimiert wird [119]. In der Zusammenschau sind B‑Zellen allerdings nicht alleinig für die Entstehung und Aufrechterhaltung der MS verantwortlich: Es gibt Hinweise dafür, dass (Gedächtnis‑)B-Zellen autoreaktive, autoproliferative [120], proinflammatorische T‑Zellen (u. a. T_H_17-Zellen) induzieren, die wiederum eine entscheidende Rolle in den Entzündungskaskaden im ZNS spielen [121–123], und dass „polymorphonuclear myeloid-derived suppressor cells“ (PMN-MDSCs) selektiv die Akkumulierung von B‑Zellen im ZNS kontrollieren [124]. Nichtsdestotrotz war es bis heute nicht möglich, ein bestimmtes Zielantigen dieser Immunzellen zu identifizieren [125]. Zusätzlich ist bislang unklar, welche Rolle die kleine Subgruppe der CD20-positiven T‑Zellen bei der MS spielt [126]. So zeigte eine Phase-2a-Studie mit dem CD20-depletierenden Antikörper Ublituximab eine Veränderung des T‑Zell-Profils bei MS-Patienten unter Therapie [127].

## Ocrelizumab: Indikationen, praktische Aspekte und Therapiehinweise

Die Indikationen von Ocrelizumab sind in Tab. 4, das Anwendungsschema in Tab. 5 und der detaillierte Infusionsablauf im Handbuch Ocrelizumab des Kompetenznetzes Multiple Sklerose [128] dargestellt: Hinsichtlich der Indikationen ist zu beachten, dass Ocrelizumab auch für die Behandlung einer sekundär chronisch-progredienten MS zugelassen ist, sofern weiterhin aufgelagerte Schübe vorliegen. Diese relativ breite Zulassung liegt ebenfalls für die hochaktive Therapie mit Cladribin, jedoch formal nicht für Fingolimod, Natalizumab und Alemtuzumab vor.

Je nach Vortherapie sollte vor Ocrelizumab-Therapiebeginn ein Sicherheitsabstand zur Vortherapie eingehalten werden. Nach Interferon‑β, Dimethylfumarat oder Glatirameracetat sollten mögliche Effekte auf Laborwerte abgeklungen sein, Teriflunomid sollte ausgewaschen und nicht mehr im Blut nachweisbar sein, bei Fingolimod beträgt der Sicherheitsabstand mindestens 4 Wochen, bei Natalizumab 6 bis 8 Wochen, bei Cladribin 6 Monate und bei Alemtuzumab 6 bis 12 Monate. Laborchemische Untersuchungen vor Ocrelizumab-Infusion sind erforderlich. Wichtig ist, dass in Einzelfällen ggf. von oben genannten Sicherheitsabständen abgewichen werden muss, basierend auf einer individuellen Risiko-Nutzen-Bewertung. Es wird ein Differenzialblutbild, ein IgG-Spiegel im Serum, CRP, Urinstatus und ggf. ein Schwangerschaftstest empfohlen. Infektiologisch sollte vor Ocrelizumab-Therapiebeginn eine Hepatitis-B/C-, HIV-, VZV-Serologie sowie bez. Tuberkulose ein Interferon-γ-Freisetzungstest (z. B. Quantiferon) erfolgen. Patienten mit ausgeheilter, nicht mehr aktiver Hepatitis B können ggf. Ocrelizumab erhalten, jedoch ist analog zu dem Vorgehen bei Rituximab eine Vorstellung in einem Leberzentrum zur Diskussion einer prophylaktischen Therapie mit Tenofovir und engmaschige Kontrollen der HBV-DNA im Blut sinnvoll [129–131]. Alle 6 Monate sollte unter Therapie das Differenzialblutbild und ein IgG-Serumspiegel kontrolliert werden. Ocrelizumab kann zu einem erniedrigten IgG-Spiegel führen [132]. Bei erhöhter Infektanfälligkeit und erniedrigtem IgG-Serumspiegel kann eine intravenöse IgG-Substitution nach internistischer Maßgabe erfolgen. Immunphänotypisierungen vor und während der Therapie sind nicht obligat, sondern fakultativ.

Vor Ocrelizumab-Infusion sollte eine Vortherapie mit 100 mg Methylprednisolon intravenös und ein Antihistaminikum verabreicht werden, um eine mögliche Infusionsreaktion zu minimieren. Das medizinische Personal, das Ocrelizumab verabreicht, sollte für die Behandlung schwerer anaphylaktischer Reaktionen geschult sein, ferner ist ein uneingeschränkter Zugang zu intensivmedizinscher Versorgung in der eigenen oder nächstliegenden Einrichtung sinnvoll. Patienten sollten nach Ocrelizumab-Infusion für mindestens 1 h nachbeobachtet werden. Eine klinisch-neurologische Behandlungskontrolle ist nach dem ersten Behandlungsmonat und im Verlauf einmal im Quartal sinnvoll. Ferner sollte nach einem Jahr Therapie eine kritische Prüfung der Therapieindikation nicht nur hinsichtlich des Effekts auf den Krankheitsverlauf, sondern auch hinsichtlich Nebenwirkungen und paraklinischer Befunde erfolgen. Eine MRT des Schädels ist 3 Monate („baseline“ nach Einsetzen der therapeutischen Wirkung) nach Therapiebeginn und im Verlauf einmal jährlich sinnvoll. Auf eine Kontrastmittelgabe kann dabei verzichtet werden [133]. Sollte es notwendig sein, die Ocrelizumab-Therapie umzustellen, ist eine 6‑ bis 12-monatige Therapiepause sinnvoll, ferner sollte eine Zytopenie möglichst abgeklungen sein.

Sollte es bei einer schubförmigen MS zu Krankheitsaktivität unter Ocrelizumab kommen, kann eine Umstellung auf eine andere hochaktive Therapie sinnvoll sein. Der Einsatz von Ocrelizumab ist bei PPMS-Patienten im frühen Krankheitsstadium mit entzündlicher Aktivität sinnvoll [134]. Da Ocrelizumab das einzige zur Behandlung der primär chronisch-progredienten MS zugelassene Medikament ist, kann bei PPMS-Progression unter Ocrelizumab mangels zugelassener Therapiealternativen keine Empfehlung zur Umstellung erfolgen, sondern die Therapie wird in den meisten Fällen fortgeführt werden. Patienten, die bereits vor Zulassung von Ocrelizumab auf Rituximab eingestellt sind und keine Krankheitsaktivität und Nebenwirkungen zeigen, können aus medizinischer Sicht die Rituximab-Therapie fortsetzen. Da dies „off label“ ist, empfiehlt sich die vorherige Einholung einer Kostenübernahmeerklärung der Krankenversicherung.

Hinsichtlich viraler Epidemien (wie z. B. aktuell SARS-CoV-2/COVID19) besteht laut einer Stellungnahme der Deutschen Multiple Sklerose Gesellschaft (DMSG) ein erhöhtes Risiko, unter Ocrelizumab zu erkranken [135]. Da bei Ocrelizumab das Infektionsrisiko unmittelbar nach Infusion am größten ist, kann eine Streckung des Intervalls individuell diskutiert werden [136] und die Indikation zur Ersteinstellung ist kritisch zu stellen. Laut Kompetenznetz Multiple Sklerose (KKNMS) besteht bei Patienten unter Ocrelizumab insbesondere in den ersten Wochen nach Anwendung eine größere Infektionsgefahr, daher sollte in dieser Zeit besonders sorgfältig auf die Vermeidung von Infektionserkrankungen geachtet werden. Bei älteren Patienten oder Patienten mit begleitenden Herz- oder Lungenerkrankungen sollte die Einleitung einer Ocrelizumab-Therapie ggf. verschoben werden [137]. Die Schweizerische Multiple Sklerose Gesellschaft empfiehlt Ocrelizumab-Patienten in der Schweiz, die in einem Gebiet mit vielen SARS-CoV-2-Fällen leben, sich so weit wie möglich zu isolieren, um ihr Infektionsrisiko zu verringern, und den Beginn einer Ocrelizumab-Therapie sorgsam abzuwägen [138]. Die Multiple Sclerosis International Federation rät Patienten unter Therapie, sich bei Verdacht auf, oder bei einer bestätigten Infektion mit SARS-CoV‑2 nach vorheriger telefonischer Absprache, beim behandelnden Arzt vorzustellen. Das Risiko einer schwerwiegenden SARS-CoV-2-Infektion wird unter Glatirameracetat, Interferon‑β oder Natalizumab als am geringsten eingeschätzt [139]. Eine verzögerte Ocrelizumab-Gabe kann auch aus Sicht des Multiple Sclerosis Trust und der Association of British Neurologists erwogen werden, um ein Infektionsrisiko zu reduzieren [140, 141].

Um Infektionskrankheiten vorzubeugen, sollten alle Patienten, die Ocrelizumab bekommen, vor Erstgabe nach den Empfehlungen der Ständigen Impfkommission (STIKO) für immunsupprimierte Patienten inkl. jährlicher Grippeschutz- und Pneumokokkenimpfung vakziniert werden. Zwischen Impfung und Ocrelizumab-Infusion sollte ein Abstand von 6 Wochen liegen, Lebendimpfungen sind unter Ocrelizumab kontraindiziert.

Ocrelizumab darf während der Schwangerschaft und Stillzeit nicht angewendet werden, weswegen eine wirksame Empfängnisverhütung unter Ocrelizumab für mindestens 12 Monate nach jeder Ocrelizumab-Gabe von der EMA empfohlen wird. Neue Daten legen nahe, dass dieser Sicherheitsabstand in Zukunft möglicherweise verkürzt werden kann [85].

## Schlussfolgerung

In der Zusammenschau hat man bei der Behandlung der Multiplen Sklerose in den letzten Jahren viel erreicht; die Zulassung von Ocrelizumab war ein weiterer wichtiger Schritt in die Richtung einer effektiven Behandlung von MS-Patienten. Dieser humanisierte anti-CD20-monoklonale Antikörper, zweimal jährlich intravenös verabreicht, zählt zu den Hochwirksamkeitsmedikamenten zur Behandlung der schubförmigen MS mit einem bislang guten Sicherheitsprofil bei halbjährlicher Anwendung.

Hervorzuheben ist, dass Ocrelizumab das erste Medikament mit Wirksamkeit bei PPMS ist. Wichtig, wie bei jeder immunmodulierenden Substanz, sind eine rigorose Pharmakovigilanz, die Analyse von Registerdaten und die Ergebnisse von Phase-4-Studien (Beispiel die in Deutschland aktuell rekrutierende CONFIDENCE-Studie [64]). Weitere mechanistische Studien zur Aufklärung der Wirkweise der CD20 B‑Zell-Depletion sind angezeigt.
